# Changes in Human Erythrocyte Exposed to Organophosphate Flame Retardants: Tris(2-chloroethyl) Phosphate and Tris(1-chloro-2-propyl) Phosphate

**DOI:** 10.3390/ma14133675

**Published:** 2021-07-01

**Authors:** Bożena Bukowska

**Affiliations:** Department of Biophysics of Environmental Pollution, Faculty of Biology and Environmental Protection, University of Lodz, Pomorska Str. 141/143, 90-236 Lodz, Poland; bozena.bukowska@biol.uni.lodz.pl

**Keywords:** flame retardants, organophosphate flame retardants, methemoglobin, reactive oxygen species, glutathione, antioxidative enzymes, hemolysis, eryptosis, human erythrocytes

## Abstract

Tris(2-chloroethyl) phosphate (TCEP) and tris(1-chloro-2-propyl) phosphate (TCPP) are the main representatives of organophosphate flame retardants (OPFRs). The exposure of humans to OPFRs present in air, water, and food leads to their occurrence in the circulation. Thus far, no report has been published about the influence of these retardants on non-nucleated cells like mature erythrocytes. Therefore, the impact of TCEP and TCPP (in concentrations determined in human blood as well as potentially present in the human body after intoxication) on human erythrocytes was evaluated. In this study, the effect of TCEP and TCPP on the levels of methemoglobin, reduced glutathione (GHS), and reactive oxygen species (ROS), as well as the activity of antioxidative enzymes, was assessed. Moreover, morphological, hemolytic, and apoptotic alterations in red blood cells were examined. Erythrocytes were incubated for 24 h with retardants in concentrations ranging from 0.001 to 1000 μg/mL. This study has revealed that the tested flame retardants only in very high concentrations disturbed redox balance; increased ROS and methemoglobin levels; and induced morphological changes, hemolysis, and eryptosis in the studied cells. The tested compounds have not changed the activity of the antioxidative system in erythrocytes. TCPP exhibited a stronger oxidative, eryptotic, and hemolytic potential than TCEP in human red blood cells. Comparison of these findings with hitherto published data confirms a much lower toxicity of OPFRs in comparison with brominated flame retardants.

## 1. Introduction

Flame retardants (FRs) are substances that have been designed to prevent the occurrence and spread of fire and are used as plasticizers. These chemicals are commonly used in the production of textiles (e.g., in baby clothing, pushchairs, car seats), furniture, and electronics, and are utilized in plastic equipment.

Some of these compounds migrate from products into the environment. For example, FRs may be released from textiles, and then may penetrate ground and surface waters [1]. FRs’ presence has been observed in indoor air and dust [2,3,4], as well as in various compartments of the environment, including surface water [5], arctic air [6], and sediments [7].

The results of various studies have indicated that children have higher FRs levels in serum [8,9] and urine [10] as compared with adults.

There are two ways for dust to get into the mouths of. The first is when toddlers swallow dust adjoined to their hands, and the second is when toddlers put objects in their mouths and swallow dust that sticks to the object [11]. It is known that exposure to FRs is potentially hazardous to children’s health and is associated with various disorders such as neurodevelopmental changes [12] and perturbations in the endocrine system [13].

**Figure 1:**
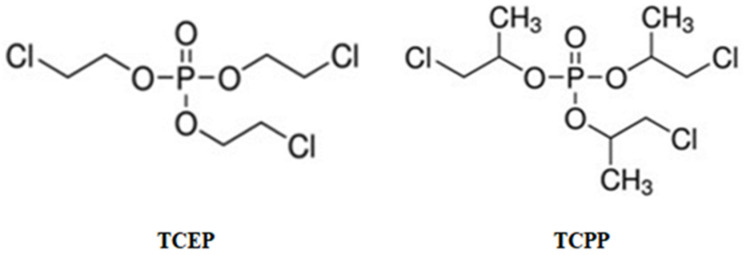
Chemical formulas of tris(2-chloroethyl) phosphate (TCEP) and tris(1-chloro-2-propyl) phosphate (TCPP).

One of the important groups of FRs is organophosphate FRs (OPFRs). OPFRs are phosphoric acid derivatives that are divided into three groups, including chlorinated OPFRs, alkyl-OPFRs, and aryl-OPFRs [14]. The synthesis of OPFRs covers about 20% of the world production of FRs [15]. Tris(2-chloroethyl) phosphate (TCEP) and tris(1-chloro-2-propyl) phosphate (TCPP) are representatives of chlorinated OPFRs (Figure 1), and are utilized in several commercial preparations [16].

Published statistical data have indicated that the use of OPFR increased as much as six times within 12 years (from 2006 to 2018) [17]. It is expected that the usage of OPFRs will reach 6 × 10^5^ tones in 2021 [18]. OPFRs are released from various products by abrasion or leaching during their usage or recycling, thus reaching the environment [19]. The common use of OPFRs results in the increase of their concentrations in dust (224 μg/g), indoor air (635 ng/m^3^), drinking water (1600 ng/L), soil (2100 ng/g), and sediments (7460 ng/g) [20]. Zhou et al. [4] reported (research conducted in Germany) that OPFRs level in the internal microenvironment was eight times higher when compared with that in the outdoor microenvironment, which suggested the presence of principal sources of these substances in the indoor microenvironment. Bekele et al. [21] determined the total concentrations of 17 OPFRs in lipids of aquatic organisms, sediments, and sea water in the concentration ranges of 21.10–3510 ng/g, 0.10–96.90 ng/g, and 0.20–28.40 ng/L, respectively. These results have unequivocally showed the hazard of trophic transfer of OPFRs into the food web. Finally, these substances were detected in marine and freshwater animals [22] and human samples, i.e., human breast milk [23], blood [24,25], urine [26], hair [27], adipose tissue, and various organs [28]. Zhao et al. [25] determined eleven OPFRs in the blood of 257 inhabitants of Shenzhen (China). They observed detectable levels of six OPFRs, including tris(2-chloroisopropyl) phosphate (TCIPP), in at least of 90% of participants, with a median concentration of TCIPP calculated as 0.71 ng/mL. The inhalation and dermal absorption of OPFRs [15] pose an essential risk to adults, while hand-to-mouth contact and breast feeding are the dominant routes of exposure for infants and young children [2]. Higher hand wipe FRs levels are when objects (e.g., electronic devices) are touched that contain high concentrations of these substances [11]. Textiles from which these compounds are emitted into the atmosphere and are transferred from hand to mouth are also an important source of retardants [1]. For this reason, new, potentially low-toxic, eco-friendly flame retardants are being developed [29,30].

TCEP production has reached 1.0 megatons globally [20]. High concentrations of TCEP have been determined in indoor dust (~2.0 × 10^5^ ng/g). Moreover, its presence in nearly all foodstuffs (max. concentration of ~30–300 ng/g or ng/L) as a human body burden has been reported [18]. Similarly to other traditional FRs, TCEP can be transferred between trophic levels, as has been determined in all food stuffs (studies in Sweden and Belgium), such as cereals, meat, fish, dairy, eggs, vegetables, and fruit, as well as sweets and beverages [31,32]. Poma et al. [32] found a higher concentration of OPFRs in fats/oils (84.40 ng/g, *w/w*) in comparison with cereals (36.90 ng/g, *w/w*) and cheese (20.10 ng/g). In turn, Zhang et al. [33] detected OPFRs in Chinese food at concentrations from 0.004 to 287 ng/g. Rice and vegetables contain the highest amounts of OPFRs. It should be noted that Chinese rice contained TCEP at 29.80 ng/g, which was 1–54 times higher than for other OPERs congeners [20]. The above findings indicate that the most consumed food in a large part of the human population is significantly contaminated with TCEP [18].

Many toxicological studies conducted in vivo on various kinds of species (short- and long-term exposure) have revealed that OPFRs have adverse developmental effects, are neurotoxic, and induce oxidative stress [14]. For example, studies on laboratory animals have demonstrated that TCEP, TCPP, and tris-(1,3-dichloro-2-propyl) phosphate (TDCIPP) have neurotoxic, immunotoxic, and carcinogenic effects [34,35,36]. European Chemicals Agency [37] suggested in the screening report that the usage of TCEP, TCPP, and tris-(1,3-dichloro-2-propyl) phosphate (TDCIPP) in materials should be restricted.

Taking into account high exposure of humans, especially children, to OPERs [11,14], their presence in the circulation [25], and the lack of experimental data on the effect of TCEP and TCPP on human erythrocytes, the impact of these substances on this blood cell type was assessed. The erythrocytes are the most abundant cells within the circulatory system. Those cells play a crucial role in the human organism as they transport oxygen and carbon dioxide. Red blood cells also play an important role in the transport of endogenous and exogenous compounds, thus they are exposed to various xenobiotics entering the human organism [38].

The aim of this study was to determine the effect of tris(2-chloroethyl) phosphate and tris(1-chloro-2-propyl) phosphate on hemolysis and eryptosis induction and hemoglobin oxidation in human erythrocytes (Figure 1). Moreover, changes in ROS and GSH levels and the activities of antioxidant enzymes, including superoxide dismutase (SOD), catalase (CAT), and glutathione peroxidase (GSH-Px), in red blood cells exposed to the above mentioned OPFRs were assessed. The effect of TCEP and TCPP at both low concentrations of 0.001 µg/mL (determined in human blood [25]) and high concentrations (which may enter human organisms only as a result of acute poisoning) was studied. The analysis of higher concentrations of the examined compounds allowed to describe the mechanism of their action in red blood cells.

## 2. Materials and Methods

### 2.1. Chemicals

Tris(2-chloroethyl) phosphate (TCEP) (purity 97%) and tris(1-chloro-2-propyl) phosphate (TCPP) ((a mixture of isomer tris(1-chloro-2-propyl) phosphate 66% and minor components: bis(1-chloro-2-propyl) (2-chloropropyl) phosphate and (1-chloro-2-propyl) bis(2-chloropropyl) phosphate)), were purchased from Sigma-Aldrich (St. Louis, MO, USA). 5,5-Dithio-bis-2-nitrobenzoic acid (DTNB), NADPH, glutathione reductase (GR), reduced glutathione (GSH), and epinephrine were bought from Sigma-Aldrich (St. Louis, MO, USA). Ethanol, chloroform, and other chemicals were purchased from Carl Roth (Roth, Germany) and POCH (Gliwice, Poland). Other chemicals used were also of the highest commercial grade available.

### 2.2. Isolation and Treatment of Human Erythrocytes

The erythrocytes were isolated from the leucocyte-buffy coat separated from blood bought in the Regional Centre of Blood Donation and Blood Treatment in Lodz, Poland. Blood was collected from anonymous healthy volunteers. All blood donation procedures were performed at the Regional Centre of Blood Donation and Blood Treatment in Lodz, Poland. 

The research was approved by the Bioethics Committee of the University of Lodz No. 1/KBBN-UŁ/I/2020-21. The investigations were agreed to by the Bioethics Committee of the University of Lodz, and no informed consent is needed for studies on buying anonymous human blood samples. All used methods were performed in accordance with the relevant guidelines and regulations.

The leukocyte-platelets buffy coat containing erythrocytes was centrifuged (600× *g*, 10 min, 4 °C) to separate red blood cells. After the sample had been centrifuged, the RBCs were found at the bottom as the precipitate. Isolated red blood cells were washed three times with Ringer buffer (5 mM KCl, 125 mM NaCl, 1 mM CaCl_2_, 1 mM MgCl_2_, 32 mM HEPES, 25 mM Tris, 10 mM glucose, pH 7.4) by centrifugation (600× *g*, 10 min, 4 °C). Hematocrit was determined using a microhematocrit centrifuge. The cells were diluted to 5% hematocrit using a Ringer buffer and incubated with TCPP and TCEP (0.001–1000 μg/mL) at 37 °C for 24 h. Earlier studies have shown that incubation of red blood cells for 48 h and 72 h caused significant changes in control erythrocytes, including hemoglobin oxidation (% metHb—2.38%, 24 h; 3.95%, 48 h; 7.12%, 72 h [39]). To eliminate the possible negative impact on the extent of the assessed changes, the time of 24 h of incubation was selected as the optimal time for this study. A wide range of concentrations of the tested compounds were used from those relevant to environmental exposure to very high ones to take into account their cumulative effects that may appear during occupational exposure.

TCEP and TCPP were dissolved in dimethyl sulfoxide (DMSO). The final concentration of DMSO in the samples was 0.4%. Owing to a longer incubation time, 24 h, antibiotics, i.e., streptomycin and penicillin (0.2%), were added to the erythrocytes suspension. We excluded the toxic effect of DMSO and antibiotics (in the concentrations used in this study) on erythrocytes [40]. Control samples consisted of erythrocytes, which were incubated with Ringer buffer and DMSO (the final concentration in the sample was 0.4%) [41].

### 2.3. Hemolysis

The absorbance of hemoglobin leaked from the cells was recognized as an indicator of erythrocytes viability [42]. The determination of hemolysis was described in the publication of Jarosiewicz and co-workers [39].

### 2.4. Quantification of Apoptosis—Analysis of Phosphatidylserine (PS) Externalization

Annexin V conjugated to fluorescein isothiocyanate (FITC) binds to phosphatidylserine (PS). As a result of oxidative stress, PS undergoes excessive externalization at the outer leaflet of the membrane bilayer. This method was described by Jarosiewicz and co-workers [43]. 

### 2.5. Morphological Changes

#### 2.5.1. Phase Contrast Microscopy

The cells (about 5% hematocrit) after 24 h of incubation with OPFRs were mixed with PBS to 0.02% hematocrit and placed in Petri dish. The photos were taken using a phase contrast microscope (Olympus, Tokyo, Japan) at a magnification of 600×.

#### 2.5.2. FSC Parameter

Control erythrocytes and the cells incubated with TCEP and TCPP were studied using flow cytometry (LSR II, Becton Dickinson, East Rutherford, NJ, USA). Detection of low angle (FSC-A) light scattering was described in the previous publication [44].

### 2.6. Hemoglobin Oxidation

Measurement and calculation of hemoglobin level were described in the previous publication of Jarosiewicz et al [39].

### 2.7. Oxidation of 6-Carboxy-2′,7′-Dichlorodihydrofluorescein Diacetate (H_2_DCFDA)

The oxidation of H_2_DCF was analyzed by flow cytometry using fluorescent stain 6-carboxy-2′,7′-dichlorodihydrofluorescein diacetate. The determination of the oxidation of 2′,7′-dichlorodihydrofluorescein was described by Jarosiewicz and co-workers [43].

### 2.8. Reduced Glutathione Level and Antioxidant Enzyme Activity

Reduced glutathione (GSH) was assessed by the method of Ellman [45]. In this method, DTNB (5,5-dithiobis-(2-nitrobenzoic acid)) is reduced by thiol compounds (mainly GSH) to yield colored 2-nitro-5-mercaptobenzoic acid, whose absorbance is measured at 412 nm. The level of GSH was presented in μmol/mL PC.

Determination of SOD was analyzed by the method of Misra and Fridovich [46]. In this method, SOD inhibits epinephrine self-oxidation in an alkaline conditions. The superoxide anion is created as an intermediate product in the reaction of colored adrenochrome formation. The activity of SOD was expressed in U/g hemoglobin. Catalase activity, including catalase and hemoglobin activity, was determined by evaluating the degradation of hydrogen peroxide. CAT activity was measured in a hemolysate at 240 nm and expressed in µmol/min/mg Hb [47]. The activity of GSH-Px was assessed by the method of Rice–Evans [48]. The tert-butyl peroxide was used as a reaction substrate. The activity of GSH-Px was expressed in µmol/min/g Hb.

### 2.9. Statistical Analysis

Statistical analysis was described by Jarosiewicz and co-workers [43]. The assays were conducted on blood from four or seven donors (four or seven experiments were performed), whereas for each donor, an experimental point was an average value of at least three replications. The results were shown as mean ± SD.

## 3. Results

### 3.1. Hemolysis

**Figure 2:**
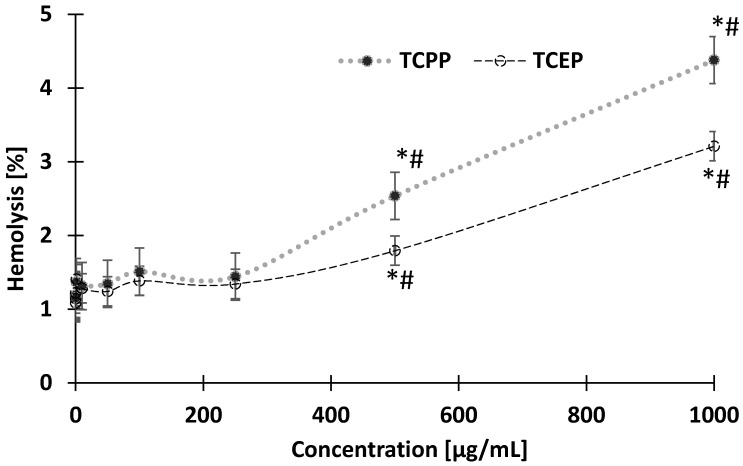
Hemolysis level in control erythrocytes and erythrocytes incubated with TCEP and TCPP in the concentrations from 0.001 to 1000 µg/mL for 24 h. * *p* < 0.05 indicates statistically significant difference from control; (#) statistically significant difference between TCPP and TCEP. One-way analysis of variance (ANOVA) and a posteriori Tukey test.

Incubation of the erythrocytes with different concentrations of TCEP and TCPP for 24 h resulted in a statistically significant increase of hemolysis (Figure 2). The tested compounds at 500 µg/mL and 1000 µg/mL caused a statistically significant increase in the above-mentioned parameters (Figure 2). It was found that TCPP at the above mentioned concentrations caused greater hemolysis than TCEP (#).

### 3.2. Quantification of Eryptosis—Analysis of Phosphatidylserine (PS) Externalization

**Figure 3:**
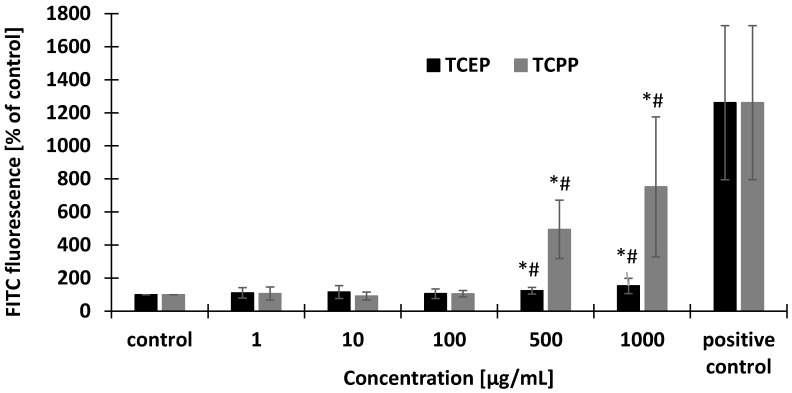
Changes in phosphatidylserine externalization (eryptosis) in control erythrocytes and erythrocytes incubated with TCEP and TCPP in the concentrations from 1 to 1000 µg/mL for 24 h. Positive control-cells incubated with valinomycin. * *p* < 0.05 indicates statistically significant difference from control; (#) statistically significant difference between TCPP and TCEP. One-way ANOVA and a posteriori Tukey test.

Changes in the FITC fluorescence were observed in the erythrocytes treated with TCPP and TECP at concentrations of 500 µg/mL and 1000 µg/mL after 24 h of incubation (Figure 3). TCPP caused stronger eryptotic changes than TCEP (#).

### 3.3. Morphological Changes of Erythrocytes, FSC Parameter

**Figure 4:**
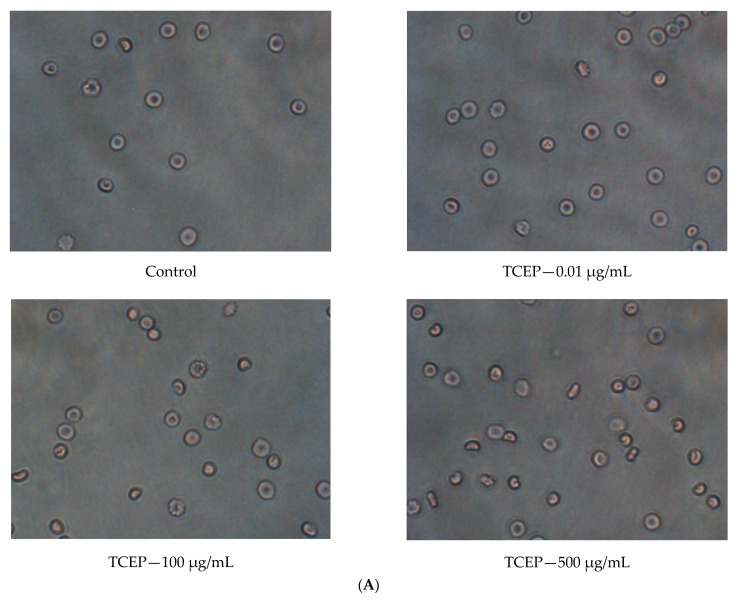

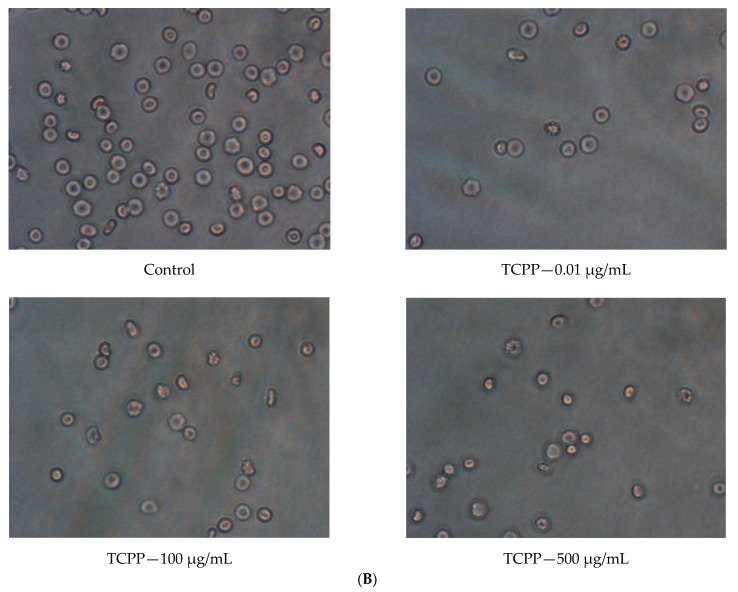
(**A**) Micrographs of control erythrocytes and erythrocytes incubated with TCEP at concentrations from 0.01 to 500 µg/mL for 24 h. (**B**) Micrographs of control erythrocytes and erythrocytes incubated with TCPP at concentrations ranging from 0.01 to 1000 µg/mL for 24 h.

TCPP and TCEP caused changes in red blood cells’ morphology. The biconcave disc shape was observed, which is typical for normal erythrocytes. The shape of erythrocytes was changed after treatment of the cells with TCPP and TCEP, but only at high concentrations of 100 µg/mL and 500 µg/mL (Figure 4A,B).

**Figure 5:**
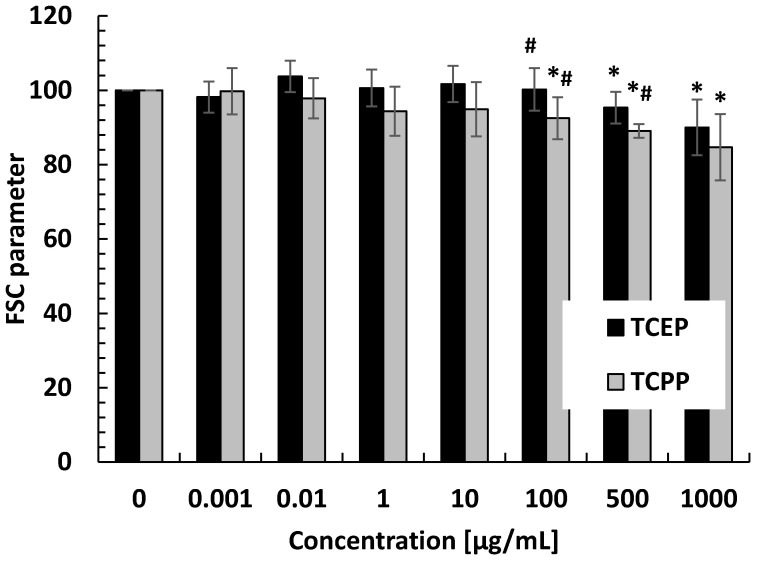
Changes in the FSC parameter in control erythrocytes and erythrocytes incubated with TCPP at concentrations ranging from 0.001 to 1000 µg/mL for 24 h. * *p* < 0.05 indicates statistically significant difference from control. (#) statistically significant difference between TCPP and TCEP.

TCEP and TCPP caused statistically significant changes in the FSC parameter (Figure 5). TCEP at 100 µg/mL and TCPP at 500 µg/mL decreased the FSC parameter in the incubated cells.

### 3.4. Hemoglobin Oxidation

**Figure 6:**
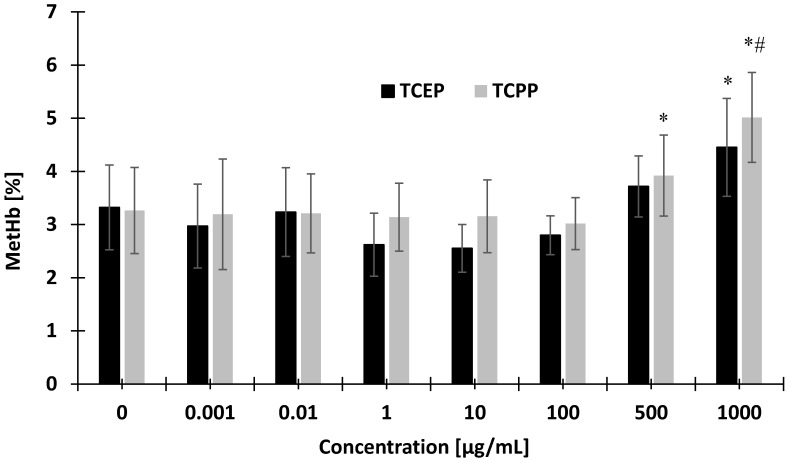
Changes in hemoglobin oxidation in control erythrocytes and erythrocytes incubated with TCEP and TCPP at concentrations ranging from 0.001 to 1000 µg/mL for 24 h. * *p* < 0.05 indicates statistically significant difference from control; one-way ANOVA and a posteriori Tukey test. (#) statistically significant difference between TCPP and TCEP.

TCEP at 1000 µg/mL and TCPP at 500 µg/mL were capable of inducing hemoglobin oxidation (Figure 6). TCPP at the highest concentration caused greater (statistically significant) hemoglobin oxidation than TCEP (#).

### 3.5. Oxidation of H_2_DCFDA—Total ROS Level

**Figure 7:**
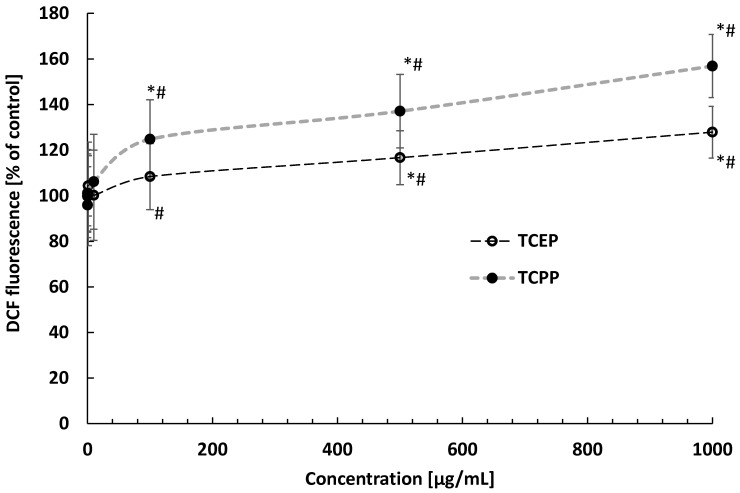
Changes in total ROS level in control erythrocytes and erythrocytes incubated with TCEP and TCPP at concentrations ranging from 0.001 to 1000 µg/mL for 24 h. * *p* < 0.05 indicates statistically significant difference from control; (#) statistically significant difference between TCPP and TCEP, one-way ANOVA, and a posteriori Tukey test.

An increase in total ROS level in the erythrocytes incubated with tested OPFRs was noted. After 24 h of incubation, an increase in total ROS level was observed in cells incubated with TCPP at 100 μg/mL and TCEP at 500 μg/mL (Figure 7).

### 3.6. The Level of Reduced Glutathione (GSH) and the Antioxidant Enzymes Activities

The cells were incubated with tested OPFRs in prehemolytical concentrations of 0.001–100 μg/mL. After 24 h of incubation, TCPP and TCEP did not change the level of reduced glutathione (Table 1).

No changes in the activity of antioxidant enzymes, i.e., SOD, GSH-Px, and CAT, were noted in the erythrocytes incubated with TCEP or TCPP in the concentrations ranging from 0.001 to 100 µg/mL for 24 h (Table 1).

## 4. Discussion

There are no results of the studies concerning the influence of OPFRs, including TCEP and TCPP, on human erythrocytes. In physiological conditions, these cells have an average life span of 120 days, but they may undergo accelerated hemolysis or eryptosis under the influence of various toxic substances, which leads to their removal from the circulation. Zhao et al. [25] determined 11 OPFRs in blood of a large nonoccupational population, and found the correlation between blood concentrations of OPFRs and the levels of sphingolipids, which play an essential role as structural components of the cell membrane, affecting the functions of the erythrocytes.

This study showed that TCEP and TCPP caused statistically significant hemolytic potential, but hemolytic changes occurred only at very high concentrations of 500 μg/mL and 1000 μg/mL, which have never been determined in the human organism. It was observed that TCPP caused greater leakage of hemoglobin from the cell when compared with TCEP at the same concentrations. The consequence of hemolysis (observed in this study) may be changes in cell morphology (Figure 4A,B) and cell size (Figure 5). Mokra et al. [49] showed a similar association in nucleated blood cells, i.e., human peripheral blood mononuclear cells (PBMCs) treated with TCEP and TCPP. They observed that TCEP and TCPP at the concentration of 1 mM and 0.5 mM, respectively, significantly decreased the viability of human PBMCs. Additionally, it was observed that TCPP, unlike TCEP, induced morphological changes of this cell type [49]. The relatively low cytotoxicity of TCEP has also been shown in other studies, including those published by Jarema et al. [50] and Cheng et al. [51].

The mature red blood cells may die as a result of programmed cell death—eryptosis. During this process, cell contraction, loss of peripheral membrane proteins, and bulging of the plasma membrane appear. Eryptosis results from local changes in the cell membrane are associated with the phosphatidylserine exposure on its outer leaflet, and it is often caused by oxidative stress. In the final step of eryptosis, red blood cells are removed by phagocytic cells and destroyed [52]. This study showed that TCEP and TCPP at their highest concentrations of 500 µg/mL and 1000 µg/mL caused a statistically significant increase in the number of apoptotic erythrocytes, while TCPP caused greater eryptosis when compared with TCEP at the same concentrations.

ROS play an important role in the induction of both apoptosis [53] and hemolysis; therefore, their level in erythrocytes was examined. An increase in ROS level was found in the tested cells incubated with both studied compounds. It was found that TCPP at 100 µg/mL caused oxidative changes, while TCEP only at its highest concentrations of 500 µg/mL and 1000 µg/mL induced ROS formation. It was also found that both tested compounds caused statistically significant increase in methemoglobin level, which was probably associated with enhanced ROS formation. The role of ROS in the induction of oxidative stress as well as death of the cells exposed to OPFRs has been described in several papers. Zhang et al. [54] showed that TCEP increased mitochondrial ROS production in liver cells (at 3.12 µg/mL), disrupted mitochondrial integrity, and caused mitochondrial dysfunction, which was associated with increased intracellular calcium ions level, decreased transmembrane mitochondrial potential, and cell cycle arrest. Similar conclusions were made by An et al. [55] analyzing the effects of TCPP on three human in vitro models, like HepG2 hepatoma cells, the A549 lung cancer cells, and the Caco-2 colon cancer cells. The authors of the study showed that TCPP at relatively high concentrations (at 49.14 µg/mL) decreased cell viability, increased ROS production, induced DNA lesions, and increased lactate dehydrogenase (LDH) leakage. Moreover, they noted that changes in cells’ viability and ROS level occurred at much lower TCPP concentrations (3.12 and 49.14 µg/mL) when compared with non-nucleated erythrocytes (changes at 100 µg/mL). Oxidative stress can result from an increase in ROS level and/or changes in the activity of the components of antioxidative system. The level of GSH and the activities of antioxidant enzymes (SOD, CAT, and GSH-Px) play an important role in the protection of red blood cells against oxidative stress. In this study, no changes in GSH level were observed in the erythrocytes incubated with TCPP and TCEP. The activities of SOD, CAT, and GSH-Px were also not changed, which showed that examined OPFRs were incapable of affecting the activity of enzymatic and nonenzymatic antioxidant defenses in human red blood cells. Contrary to organophosphorus compounds, studies conducted by Jarosiewicz et al. [41] indicated a very strong impact of brominated flame retardants (BFRs) on the activity of antioxidant enzymes in human erythrocytes. These authors showed a decrease in the activity of SOD, CAT, and GSH-Px under the influence of BFRs, while in the case of tetrabromobisphenol A (TBBPA), changes occurred even at a concentration of 10 µg/mL.

As mentioned above, studies concerning other flame retardants, like BFRs, indicated their incomparable higher toxicity in comparison with TCEP and TCPP towards human blood cells, including erythrocytes. Jarosiewicz et al. [43] assessed the effect of BFRs on the formation of ROS in human erythrocytes and showed that these substances even at the concentration of 0.0001 µg/mL (1 h of incubation) exhibited a strong oxidative effect. The study by Park et al. [56] also showed that BFRs, and mainly TBBPA, strongly induced ROS formation. They noted that TBBPA even at 10 μM caused an increase in the activation of proinflammatory factors. In another study, BFRs have been shown to exert a very strong ability for hemoglobin oxidation and induction of death of red blood cells, i.e., hemolysis [39]. In another study, Jarosiewicz et al. [43] revealed that BFRs at 10 µg/mL strongly induced eryptosis, i.e., caused changes in phosphatidylserine externalization and caspase-3 activation. Włuka et al. [57] examined cytotoxic potential and oxidative properties of BFRs in human PBMCs. They observed that BFRs at relatively low concentrations decreased PBMCs’ viability and depleted the intracellular ATP level (from 5 µg/mL) as well as induced ROS (including hydroxyl radical) formation and caused oxidative damage to proteins and lipids (from 0.0001 µg/mL). The above findings, compared with the results obtained in this study, clearly show a much stronger toxic potential of BFRs in comparison with OPFRs in human blood cells.

However, it should be remembered that the weak toxic effect of phosphorus retardants observed in in vitro tests on erythrocytes and other cells discussed in this work (e.g., PBMCs) does not imply the toxicological safety of these compounds. The long-term effects of low doses of TCEP and TCPP accumulating in the kidneys and liver should also be considered. Additionally, these compounds may exert carcinogenic potential [18]. It should be emphasized that this study compares the effect of two phosphorus retardants on human blood cells and has the character of basic/mechanistic research.

Generally, the conducted research has shown that TCPP revealed stronger eryptotic, hemolytic, and oxidative potential than TCEP. It is likely that the main parameter determining the toxicity of this xenobiotic is its lipophilicity, associated with a value of its partition coefficient (K_o/w_). The octanol/water partition coefficient (K_o/w_) for TCPP and TCEP is 2.55 and 1.44, respectively, which may explain a higher toxic potential of TCPP in comparison with TCEP [58].

It is also worth mentioning that there are three methyl groups attached to oxygen atoms in the TCPP molecule. Presumably, this may be related to its stronger adverse effects in comparison with TCEP, which were observed in this study. The results have shown that toxic potential is closely related to the presence of a methyl group in the aromatic ring of a specific compound. Bukowski et al. [59] as well as the previously mentioned author, Mokra et al. [49], confirmed this thesis. Bukowski et al. [59] showed that TCEP and TCPP induced DNA damage in PBMCs, while TCPP caused DNA lesions at 10-times lower concentration (100 µM) than TCEP. Moreover, Chu et al. [60] studied other OPFRs and suggested that the toxicity of alkyl-OPFRs is highly associated with their molecular structures. These authors investigated five alkyl-OPFRs, including trimethyl phosphate (TMP), triethyl phosphate (TEP), dibutyl phosphate (DnBP), tri-n-propyl phosphate (TPrP), and tributyl phosphate (TBP). They observed that ROS and malondialdehyde (a marker of lipid peroxidation) levels as well as SOD activity were significantly elevated in cells of *Chlorella pyrenoidosa* exposed to the longest chain length compound—tributyl phosphate.

The results of toxicological studies concerning the effect of examined OPFRs on blood cells are consistent. Regardless of the kind of cells tested (nucleated or non-nucleated), changes in the examined parameters are observed only at high concentrations of TCEP and TCPP. At high concentrations, OPFRs, especially TCPP, disturb redox balance in human erythrocytes, which leads to eryptosis and hemolysis, and thus might result in accelerated removal of red blood cells from circulatory system.

## 5. Conclusions

The findings of this work show for the first time the mechanism of action of tris(2-chloroethyl) phosphate (TCEP) and tris(1-chloro-2-propyl) phosphate (TCPP) in non-nucleated cells like human mature erythrocytes. TCEP and TCPP increased ROS levels, but did not change the activity of the antioxidant enzymes, such as SOD, CAT, or GSH-Px, nor did it alter the GSH level. An increase in methemoglobin level, hemolysis, and eryptosis under the influence of TCEP and TCPP was also observed. A comparison of the actions of TCEP and TCPP showed that TCPP exhibited a stronger oxidative, eryptotic, and hemolytic potential in erythrocytes. Changes in the parameters studied occurred at OPFRs concentrations, which are not determined in the human body as a result of environmental or occupational exposure. The results of this study indicated the low toxicity of the studied OPFRs towards human red blood cells.

## Figures and Tables

**Table 1:** The level of GSH and the activities of SOD, CAT, and GSH-Px in control erythrocytes and erythrocytes incubated with TCEP or TCPP at concentrations ranging from 0.001 to 1000 µg/mL for 24 h.

OPFR [µg/mL]	GSH μmol/mL PC	SOD (U/g Hb)	CAT (µmol/min/mg Hb)	GSH-Px (µmol/min/g Hb)
TCEP control	1.04 ± 0.48	2444.84 ± 855.23	195.15 ± 28.64	33.38 ± 7.12
0.001	1.09 ± 0.33	2798.34 ± 361.01	200.99 ± 5.56	34.61 ± 6.29
0.01	1.10 ± 0.40	2789.14 ± 283.05	212.46 ± 9.35	31.47 ± 7.65
1	1.03 ± 0.46	2857.90 ± 963.81	193.41 ± 27.28	29.84 ± 6.06
10	0.98 ± 0.41	2381.96 ± 862.75	186.90 ± 15.50	31.20 ± 5.92
100	1.02 ± 0.49	2577.72 ± 973,40	194.87 ± 20.52	31.36 ± 5.25
TCPP control	1.03 ± 0.38	2365.87 ± 922.39	198.24 ± 25.31	30.39 ± 7.05
0.0001	0.96 ± 0.43	2381.95 ± 358.62	200.98 ± 5.56	33.84 ± 6.21
0.01	0.98 ± 0.34	2394.33 ± 209.11	212.46 ± 9.35	35.63 ± 8.94
1	0.99 ± 0.34	2114.02 ± 676.54	189.03 ± 33.70	31.42 ± 7.95
10	0.98 ± 0.36	2257.40 ± 633.55	196.42 ± 15.49	30.58 ± 7.61
100	0.92 ± 0.37	2578.06 ± 982.79	175.55 ± 24.88	34.77 ± 4.94

## Data Availability

The data presented in this study are available on request from the corresponding author.

## Abbreviations

CAT activity	catalase and hemoglobin activity
DCF	dichlorofluorescein
DTNB	5,5-dithiobis(2-nitrobenzoic) acid
FITC	fluorescein isothiocyanate
FRs	flame retardants
GSH	reduced glutathione
GSH-Px	glutathione peroxidase
H_2_DCFDA	6-carboxy2′,7′-dichlorodihydrofluorescein diacetate
MetHb	methemoglobin
OPFRs	organophosphate flame retardants
PBMCs	peripheral blood mononuclear cells
PS	phosphatidylserine
ROS	reactive oxygen species
SOD	superoxide dismutase
TCEP	tris(2-chloroethyl) phosphate
TCPP	tris(1-chloro-2-propyl)
